# Exogenous spermidine-induced changes at physiological and biochemical parameters levels in tomato seedling grown in saline-alkaline condition

**DOI:** 10.1186/s40529-014-0058-2

**Published:** 2014-08-01

**Authors:** Yi Zhang, Li Zhang, Xiao-Hui Hu

**Affiliations:** 1 College of Horticulture, Northwest Agricultural & Forest University, Yangling, 712100 China; 2 College of Horticulture, Shanxi Agricultural University, Taigu, 030801 China; 3 Key Laboratory of Protected Horticultural Engineering in Northwest, Ministry of Agriculture, Shaanxi Yangling, 712100 China

**Keywords:** Tomato, Saline-alkaline stress, Spermidine, Nitrogen metabolism, Antioxidant enzyme, Osmoregulation substance

## Abstract

**Background:**

Tomato is one of the most popular vegetables, and middle tolerance for salt stress. Spermidine (Spd) has an important role in plant defense mechanisms against abiotic stress; however, relatively few data are available regarding Spd in responses of tomato to saline-alkaline stress. The effect of 0.25 mmol/L Spd on some physiological parameters of two tomato cultivars grown in 75 mmol/L saline-alkaline solutions were studied. Two cultivars are cv. Jinpeng chaoguan which is a highly salt-tolerant ecotype and cv. Zhongza No. 9 which is more salt-sensitive ecotype.

**Results:**

Saline-alkaline stress upset nitrogen metabolism, induced the antioxidant enzyme activities, and accumulated much more reactive oxygen species (ROS) and osmoregulation substances in two tomato cultivars leaves. Under saline-alkaline stress condition, Spd-treated seedlings accumulated more osmoregulation substances and had greater activities of antioxidative enzymes. Exogenous Spd counteracted the stress-induced increase of contents of malondialdehyde and ammonium, glutamate dehydrogenase activity, and decreased in nitrate, nitrate reductase, nitrite reductase, glutamine synthetase, glutamate synthase, glutamate oxaloacetate transaminase, and glutamate pyruvate transaminase activities. Additionally, the effect of Spd was more significantly in salt-sensitive cultivar ‘Zhongza No. 9’.

**Conclusions:**

Overall, exogenous spermidine can attenuate negative effects of saline-alkaline stress on tomato seedlings which effects may depend on the plant species, and even cultivars.

**Electronic supplementary material:**

The online version of this article (doi:10.1186/s40529-014-0058-2) contains supplementary material, which is available to authorized users.

## Background

Saline-alkaline condition imposes a major abiotic stress on crops and represents an important limiting factor of productivity. It has been estimated that one-third of the world’s irrigated land is unsuitable for crops due to its saline condition (Frommer et al. [1999]; Wasti et al. [2012]). Plant responses to mixed salt and alkali stress are more complex than their responses to either simple salt or alkali stress (Shi and Sheng [2005]; Yang et al. [2007]) and there have been few studies of complex neutral and alkaline salt stress. In general, plant metabolism is altered and a range of defense mechanisms are activated in response to abiotic stress, presumably to compensate for the changed environmental conditions (Wasti et al. [2012]). Stress could induce excessive generation of reactive oxygen species (ROS) including superoxide anion (O_2_^−^), hydrogen peroxide (H_2_O_2_) and hydroxyl radical (HO^-^), which could cause deterioration of membrane lipids, proteins and nucleic acids, leading to increased membrane leakage of solutes (Shehab et al. [2010]; Huang et al. [2013]). One stress-defense mechanism in plants is the accumulation of compatible osmolytes (Shahba et al. [2010]), which can also be induced or enhanced by the application of chemicals to the plant (Rhodes et al. [1999]).

Polyamines are low-molecular-weight aliphatic amines with important functions in growth, cell division, DNA replication, and protein synthesis (Roychoudhury et al. [2011]). Spermidine (Spd), spermine, and putrescine are major polyamines in plants that act as second messengers, mediating responses to various environmental stressors. These stressors include osmotic stress, changes in salinity, drought conditions, and exposure to ozone, heavy metals, and ultraviolet light (Groppa and Benavides [2008]). Compared with other type of polyamine, Spd could more effectively alleviate the adverse impacts of salinity-alkalinity (Hu et al., [2012]).

Despite research efforts, little is known about the physiological functions of exogenous Spd in response to salt-alkali mixed stress. The objective of this work was to determine effects of Spd under saline-alkaline conditions in two tomato cultivars with different salinity tolerance. The response in terms of nitrogen metabolism, antioxidant enzyme activities, contents of ROS and osmoregulation substances were evaluated in order to evaluate the role of Spd in promoting tomato plants tolerance to saline-alkaline stress.

## Methods

### Plant materials and treatments

Two tomato (*Solanum lycopersicum*) cultivars were used in the study. cv. Jinpeng chaoguan is a highly salt-tolerant ecotype, while cv. Zhongza No. 9 is more salt-sensitive ecotype (Hu et al. [2012]). Tomato seeds were surface-sterilized with 4% (v/v) sodium hypochlorite, rinsed with distilled water, soaked in distilled water for 6 h at 26°C, and transferred to sterile moist Whatman No. 1 filter paper which moistened with distilled water in Petri plates. The plates were maintained in the dark at 26°C for germination. Uniformly germinated seeds were selected and cultivated in polystyrene trays filled with complex organic substrates and placed in a greenhouse with an average temperature of 26-30°C during the day and 16-18°C at night, a 16-h light (600-800 μmol · photons/m^2^ · s) followed by an 8-h dark photoperiod, and a 50-90% relative humidity. When the third leaves were fully expanded, we transplanted all of the seedlings into rectangular hydroponic containers containing continuously aerated half-strength Hoagland’s nutrient solution. When tomato seedlings were at the sixth true leaves stage, the seedlings were treated with the following treatments: (1) control (CK), half-strength Hoagland’s nutrient solution cultivation, (b) saline-alkaline treatment, tomato seedlings were exposed to half-strength Hoagland’s nutrient solution cultivation contain 75 mmol/L saline-alkaline solution (NaCl:Na_2_SO_4_:NaHCO_3_:Na_2_CO_3_ = 1:9:9:1), (c) saline-alkaline plus Spd treatment, tomato seedlings were exposed to half-strength Hoagland’s nutrient solution cultivation contain 75 mmol/L saline-alkaline solution and sprayed with 0.25 mmol/L Spd (Sigma-Aldrich, St. Louis, MO, USA). The experiment took place in a greenhouse covered totally with polycarbonate sheets and located at the horticultural experimental station of Northwest Agriculture & Forestry University, China. The experiment design included 3 treatments of each cultivar, totally being 6 treatments with 3 times under the same conditions. Each treatment has 3 containers, and each container includes 12 plants every time. The sixth leaves were harvested 4 days after the treatment for analysis different indexes.

### Analyses of NO_3_^−^-N and NH_4_^+^-N levels

Leaf samples were dried at 75°C until constant weight was obtained. The dried material (200 mg) was ground to a powder and extracted in 10 mL of distilled water for 2.5 h. Contents of NO_3_^−^-N and NH_4_^+^-N were determined according to the method of Cataldo et al. ([1975]) and Krom ([1980]), respectively.

### Assays of nitrogen metabolism enzymes

Nitrate reductase (NR), nitrite reductase (NiR), Glutamate dehydrogenase (GDH) and Glutamate synthetase (GOGAT) activities were estimated according to the methods of Gangwar and Singh ([2011]). Glutamine synthetase (GS) activity was measured using an adaptation of Lillo’s method (Lillo [1984]). The activities of glutamate oxaloacetate transaminase (GOT) and glutamate pyruvate transaminase (GPT) were measured with the method described by Liang et al. ([2011]).

### Antioxidant enzyme activity, H_2_O_2_,O_2_^−^ and malondialdehyde assay

Superoxide dismutase (SOD) activity was assayed as previously described by Dhindsa et al. ([1981]). One unit of activity was defined as the amount of enzyme required to inhibit the reduction of nitro blue tetrazolium chloride by 50% at 560 nm. Peroxidase (POD) activity was assayed as previously described (Kochba et al. [1977]). One unit of activity was defined as the amount of enzyme required to increase absorbance by 0.1 absorbance units at an optical density of 470 nm per min. Catalase (CAT) activity was assayed as described by Dhindsa et al. ([1981]). One unit of activity was defined as the amount of enzyme required to decrease 0.1 absorbance units at an optical density of 240 nm per min. Contents of H_2_O_2_ and O_2_^−^ were detected in leaves as described by Orozco-Cardenas et al. ([2001]). The content of malondialdehyde (MDA) was measured according to the method of Xu et al. ([2008]).

### Measurements of proline, soluble sugar, and soluble protein

The proline content was estimated following the method of Bates et al. ([1973]). Soluble sugars were estimated by the anthrone reagent method using glucose as the standard (Yemm and Willis [1954]). Protein was determined according to the method of Bradford ([1976]) using bovine serum albumin as a standard.

### Statistical analysis

All data presented are the mean values. All experiments were conducted using three replicates at least. All data were statistically analyzed by the analysis of variance (ANOVA) with SAS software (Version 8.1; SAS Institute, Cary, NC, USA)) using Duncan’s multiple range test at the 0.05 level of significance.

## Results and discussions

### The effect of Spd on NO_3_^−^-N and NH_4_^+^-N content

**Figure 1:**
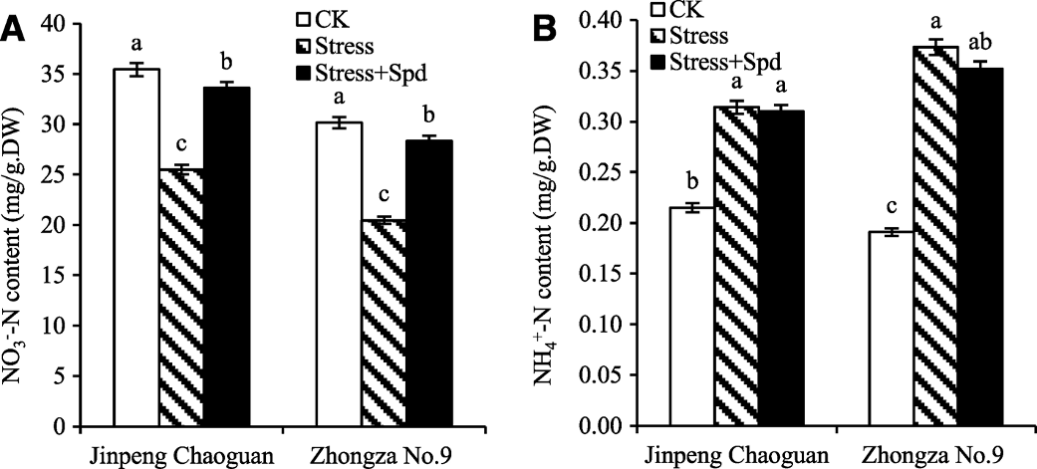
**Effects of exogenous Spd on contents of NO**_**3**_^**−**^**-N (A) and NH**_**4**_^**+**^**-N (B) in leaves of tomato seedlings under salinity-alkalinity mixed stress (nutrient solution with 75 mmol/L saline-alkaline solution; NaCl:Na**_**2**_**SO**_**4**_**:NaHCO**_**3**_**:Na**_**2**_**CO**_**3**_ **= 1:9:9:1).** Data represent means of three replicates. Bars represent the mean ± SE (*P* < 0.05) of at least three independent experiments.

Nitrogen, required in great quantities by plants, is an essential macronutrient and involved in the biochemistry of coenzymes, PAs, photosynthetic pigments, secondary metabolites, and so on. Although there are several forms of nitrogen, nitrate (NO_3_^−^) is considered to be the most available form of nitrogen in higher plants (Gajewska and Sklodowska [2009]), and it accumulates in vacuoles where it is innocuous. However, NO_3_^−^ is only a storage form of nitrogen and must be reduced to ammonium (NH_4_^+^) before it can be incorporated into organic compounds. The content of NO_3_^−^-N and NH_4_^+^-N in leaves of two tomato cultivars were clearly affected by saline-alkaline or saline-alkaline plus Spd (Figure 1). Under salinity-alkalinity stress, NO_3_^–^-N content in both cultivars leaves significantly decreased and ‘Jinpeng chaoguan’ was higher NO_3_^–^-N levels than ‘Zhongza No. 9’, while NH_4_^+^-N content in both cultivars leaves significantly increased (*P* < 0.05), and ‘Jinpeng chaoguan’ was lower NH_4_^+^-N levels than ‘Zhongza No. 9’. Although the change of the NO_3_^−^-N and NH_4_^+^-N contents occurred in both cultivars, the amplitude of change was higher in ‘Zhongza No. 9’ than in ‘Jinpeng chaoguan’. The NO_3_^–^-N decrease may be attributed to an uptake competition between NO_3_^–^ and other existing anions such as Cl^–^, SO_4_^2–^ and CO_3_^2–^, which may have unfavorable influences on plasma membrane permeability. The content of NH_4_^+^-N increased, perhaps from aggravated ion toxicity, imbalance of osmotic regulation, and/or N metabolic disturbance from the salinity-alkalinity stress. While exogenous Spd treatment increased NO_3_^−^-N content and reduced the NH_4_^+^-N content in both cultivars leaves compared to that in only stressed leaves. Overall, ‘Zhongza No. 9’ showed more sensitivity to both salinity-alkalinity stress and exogenous Spd; however, no significant differences in the NH_4_^+^-N content of ‘Jinpeng chaoguan’ seedlings grown with exogenous Spd.

### The effect of Spd on activities of nitrogen metabolism enzymes

**Figure 2:**
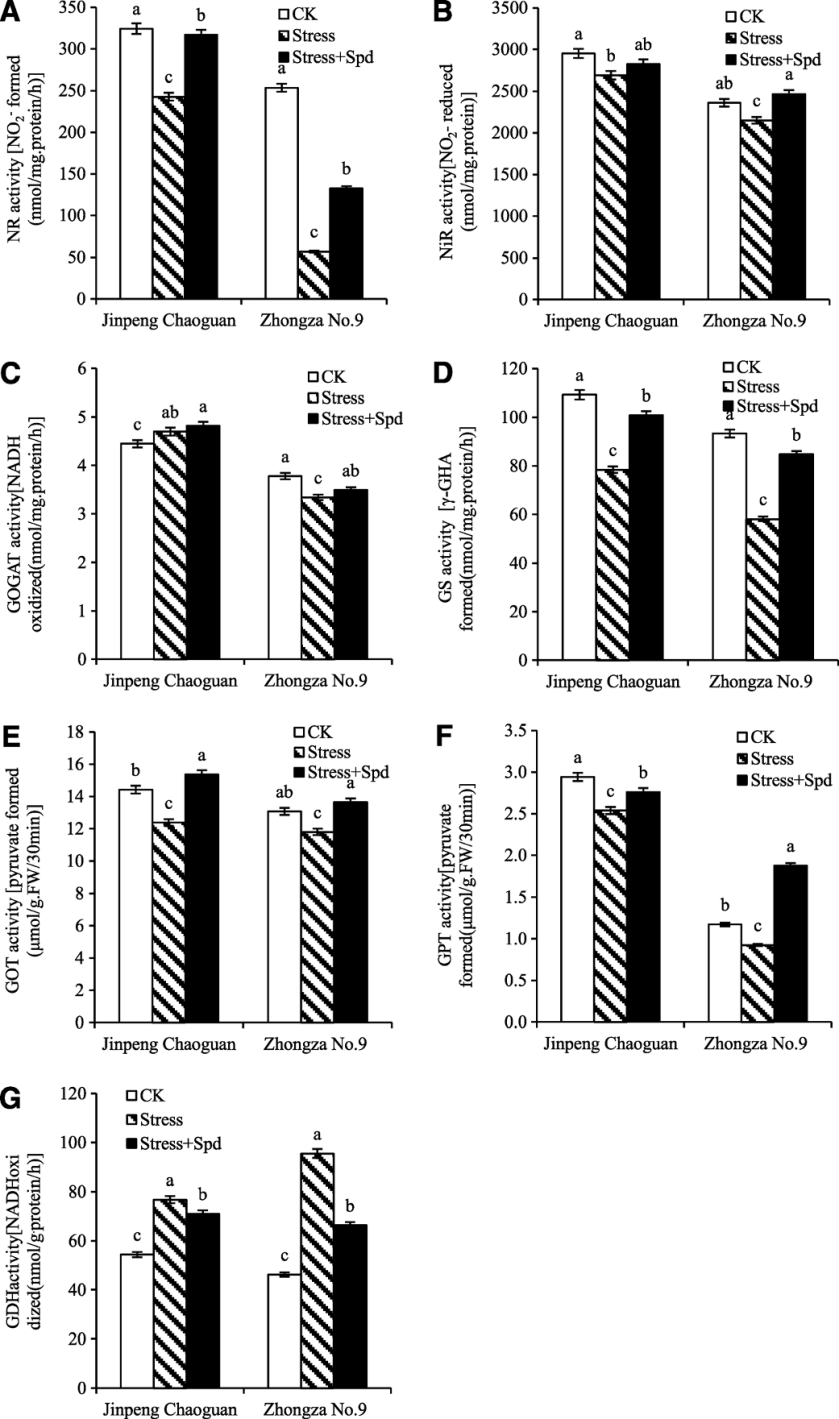
**Activities of nitrogen metabolism enzymes in leaves of tomato seedlings grown in nutrient solution or in 75 mM saline-alkaline solution (nutrient solution with 75 mmol/L saline-alkaline solution;NaCl:Na**_**2**_**SO**_**4**_**:NaHCO**_**3**_**:Na**_**2**_**CO**_**3**_ **= 1:9:9:1) with or without 0.25 mmol/L Spd.** Data represent means of three replicates. Bars indicate ± SE (*P* < 0.05). **A** delegated NR activity, **B** delegated NiR activity, **C** delegated GOGAT activity, **D** delegated GS activity, **E** delegated GOT activity, **F** delegated GPT activity, **G** delegated GDH activity. Bars represent the mean ± SE (*P* < 0.05) of at least three independent experiments.

The process of the transformation of NO_3_^−^ to NH_4_^+^ involves the successive enzymes catalysis (NR and NiR), meanwhile, NR is the rate-limiting enzyme of nitrogen assimilation. Therefore, the activity of NR can be used as an indicator of the extent of plant nitrogen metabolism. Glutamine synthetase (GS), GOGAT and GDH are the main enzymes involved in the conversion of inorganic nitrogen to organic nitrogen and in reducing ammonia toxicity (Dev and Herbert [2002]). The GS-GOGAT pathway can convert excess NH_4_^+^ into glutamic acid under drought and salt stress conditions. Ammonium ions are rapidly incorporated into organic compounds by the action of GS (Masclaux-Daubresse et al. [2006]). The activities of the NR, NiR, GOGAT, GS, GDH, GOT, and GPT in all treatments are shown in Figure 2. Salinity-alkalinity stress caused a reduction in activities of NR, NiR, GS, GOT, GPT and caused an increase in GDH activity of both cultivars. Meanwhile, the amplitude of reduction of NR, GS and GPT activities was higher in ‘Zhongza No. 9’ than in ‘Jingpeng chaoguan’, the amplitude of reduction of GOT activity was lower in ‘Zhongza No. 9’ than in ‘Jingpeng chaoguan’. Under salinity-alkalinity stress, the GOGAT activity increased in ‘Jingpeng chaoguan’ and reduced in ‘Zhongza No. 9’. In this study, leaves from plants grown under saline-alkaline stress had less NO_3_^−^-N and more NH_4_^+^-N (Figure 1), accompanied by lower NR and NiR activities (Figure 2A and B) than that in leaves from non-stressed plants. Thus, it was suggested that lower levels of NO_3_^−^-N and reduced NR and NiR activity are early symptoms of salinity-alkalinity stress. The increase in NH_4_^+^-N under saline-alkaline stress may be attributable to aggravating ion toxicity, imbalance of osmotic regulation, or a stress-induced nitrogen metabolic disturbance. The reduced activities of NR and NiR in stressed plants may have minimized the toxicity of excess NH_4_^+^ (Wang et al. [2007]) by inhibiting the conversion of NO_3_^−^-N to NH_4_^+^-N and reducing NO_3_^−^-N uptake. It also may be that rapid assimilation of NH_4_^+^ through the GS/GOGAT pathways was stimulated during salt stress. In this study, the results showed that the activities of GOT, GPT, GOGAT and GS were significantly suppressed under only saline-alkaline stress comparing with control (Figure 2C,D,E and F). This suggests that during saline-alkaline stress, NH_4_^+^competition and ammonia assimilation are mainly via the GDH pathway. In this experiment, saline-alkaline stress induced the activity of GDH (Figure 2G), which is similar to results obtained from crops (Ramanjulu et al. [1994]; Wang et al. [2007]). An increase in GDH activity can be used to catalyze the reversible reaction of glutamate from NH_4_^+^-N (Skopelitis et al. [2006]).

Compared with the only salinity-alkalinity stress, application of Spd significantly promoted NR, GS, GOT, GPT activities in two tomato cultivars, and inhibited GDH activity and had relatively little effect on leaf GOGAT activities in two tomato cultivars (Figure 2). Spd had more effects on nitrogen metabolism enzymes activities in ‘Zhongza No. 9’, except for GOT activity (Figure 2). Changes in forms of nitrogen and the particular complement of metabolic enzymes present can reflect a plant’s adaptation of nitrogen metabolism to stress (Gangwar and Singh, [2011]). The experiment indicates that exogenous Spd partly counteracted stress-induced increases in NH_4_^+^-N levels and GDH activity, as well as increased in NO_3_^−^-N content and the activities of NR, NiR, GS, GOGAT, and GOT in tomato seedling leaves under saline-alkaline stress. Spd may act as a kind of multi-functional signaling molecule that can activate a variety of defense reactions, resulting in maintenance of normal metabolism and enhanced resistance (Groppa and Benavides [2008]). The positive effects of exogenous Spd on plant nitrogen metabolism may also include further alleviation of saline-alkaline-resulted injuries. The Spd-induced resistance to saline-alkaline stress may be associated with the conversion of Spd to putrescine or spermine. Our previous results showed that exogenous Spd promoted the conversion of free putrescine to free Spd and spermine under salinity-alkalinity stress (Hu et al., [2012]), which suggested that exogenous Spd treatment can regulate the metabolic status of polyamines caused by salinity-alkalinity stress, and eventually enhance tolerance of tomato plants to salinity-alkalinity stress. Besides, some differences in the functions of Spd among cultivars of a given species exist. However, based on our observations, exogenous Spd improves stressed plants more than a control, especially in ‘Zhongza No. 9’ which is a sensitivity cultivar. This result suggested that under saline-alkaline stress, the effects of exogenous Spd on nitrogen metabolism may depend on the plant species, and even cultivars.

### Changes in antioxidant enzymes, H_2_O_2_ , O_2_^−^, and MDA contents in leaves

**Figure 3:**
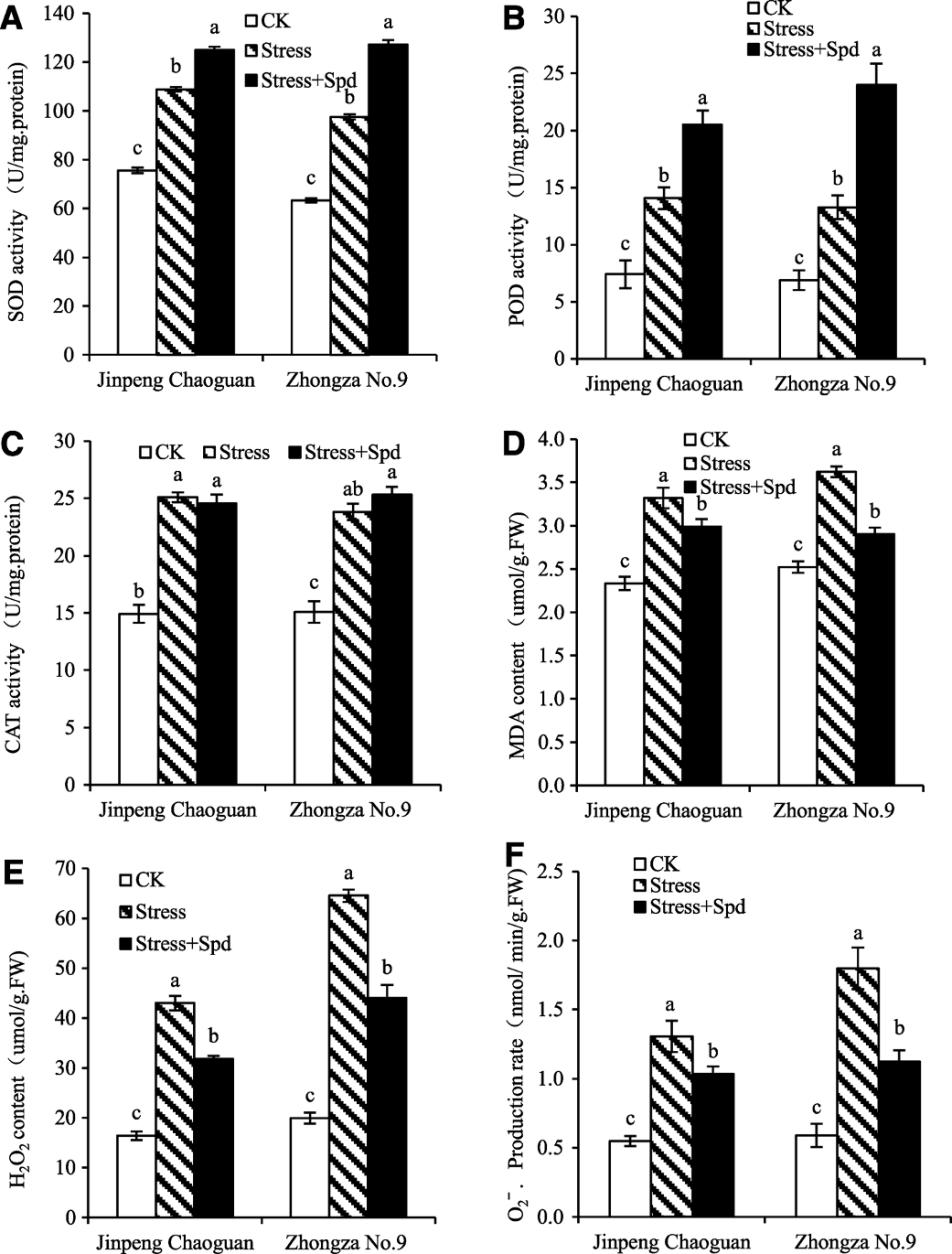
**Effects of exogenous Spd on antioxidant enzyme activities and MDA content and ROS in leaves of tomato seedlings grown in nutrient solution or in 75 mM saline-alkaline solution (nutrient solution with 75 mM saline-alkaline solution; NaCl:Na**_**2**_**SO**_**4**_**:NaHCO**_**3**_**:Na**_**2**_**CO**_**3**_ **= 1:9:9:1) with or without 0.25 mM Spd. (A)** delegated SOD activity; **(B)** delegated POD activity; **(C)** delegated CAT activity; **(D)** delegated MDA content; **(E)** delegated H_2_O_2_ content; **(F)** delegated **O**_**2**_^−^content. Bars represent the mean ± SE (*P* < 0.05) of at least three independent experiments.

Antioxidative enzymes like SOD, POD and CAT play a significant role in conferring tolerance of abiotic stress. In this study, saline-alkaline treatment elicited significant increases in the activities of SOD, POD, and CAT (*P* < 0.05) and resulted in greater accumulation of H_2_O_2_, O_2_^−^ and MDA compared with the levels in leaves of control plants for two tomato cultivars leaves (Figure 3). This tendency is more obviously in ‘Zhongza No. 9’ than in ‘Jingpeng Chaoguan’, except for CAT activity and MDA content. Application of Spd to stressed plants significantly (*P* < 0.05) increased SOD and POD activities and decreased reactive oxygen species (ROS) and MDA contents compared to the levels in plants treated only with saline-alkaline stress (Figure 3). However, Spd had no significantly effect on CAT activity. In a word, we found that exogenous Spd efficiently alleviated saline-alkaline stress in tomato seedlings, as assessed and by enzymatic activities and ROS content. These results indicate that Spd enhances saline-alkaline tolerance in tomato seedlings by efficiently scavenging ROS.

### The effect of Spd on osmoregulation substance

**Figure 4:**
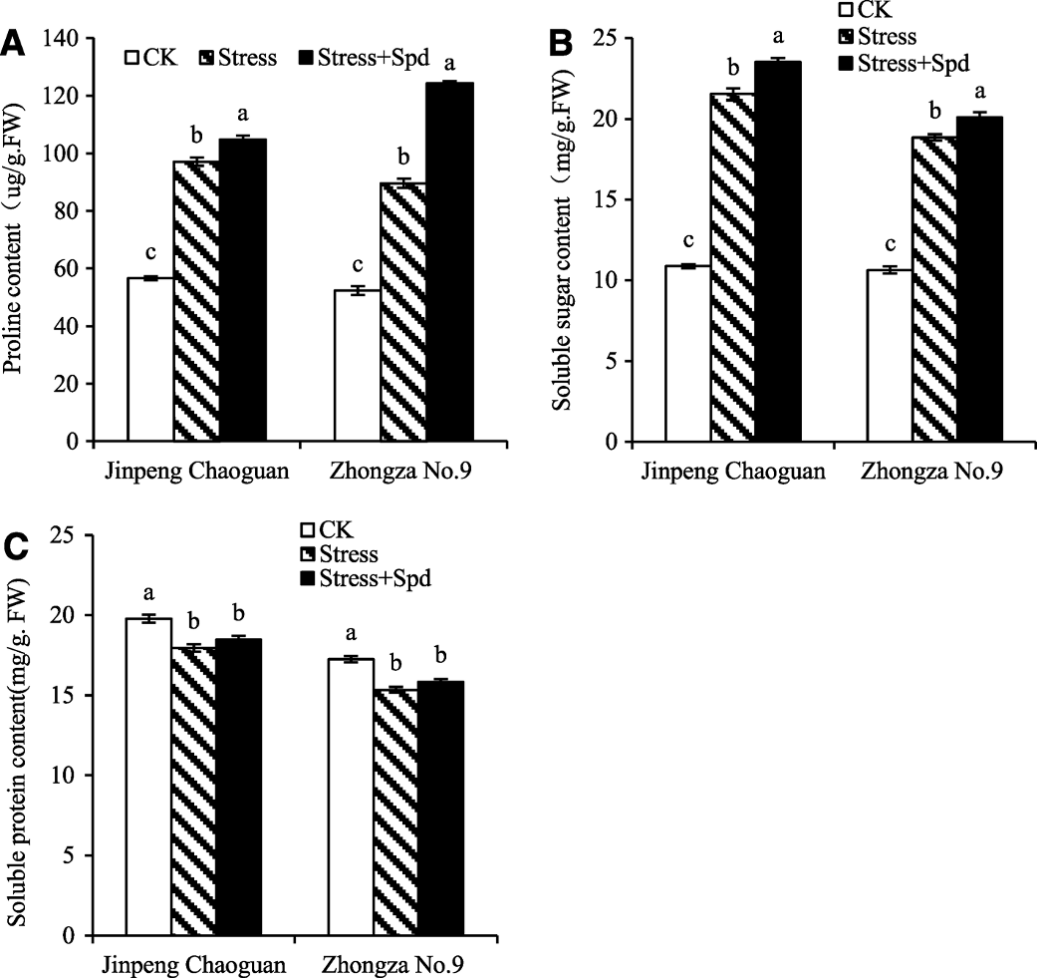
**Effects of exogenous Spd on osmoregulatory substances in leaves of tomato seedlings under salinity-alkalinity mixed stress (nutrient solution with 75 mmol/L saline-alkaline solution; NaCl:Na**_**2**_**SO**_**4**_**:NaHCO**_**3**_**:Na**_**2**_**CO**_**3**_ **= 1:9:9:1).** Bars represent the mean ± SE (*P* < 0.05) of at least three independent experiments. **(A)** delegated proline content; **(B)** delegated soluble sugar content; **(C)** delegated soluble protein content.

The accumulation of proteins in plants under saline conditions may also provide a storage form of nitrogen that is reutilized when stress is over and plays a role in osmotic adjustment. In this study, the proline and soluble sugar contents were much higher, and the soluble protein content was much lower, in leaves of two tomato cultivars subjected to saline-alkaline solution compared with the content in control plants (Figure 4) in agreement with the results of Wasti et al. ([2012]). This may have been due to enhancement of the pathway for synthesis of proline from glutamine, or by inducing other amino acids that can be transformed to proline. Under salinity-alkalinity stress, proline not only acts as an osmolyte that can prevent cell dehydration, but is also a protective agent of many biological macromolecules and an ROS scavenger. Our finding that the leaf soluble protein level decreased in response to stress agrees with the results of Abdel-Latef ([2005]), and has been suggested to be due to either the inhibition of NR activity under stress (Undovenko [1971]) or to protein synthesis (Kong-Ngern et al. [2005]). In this experiment, these substances increased in stressed leaves treated with Spd compared with the content in the stress-only plants (Figure 4). It has been suggested that Spd may inhibit polysaccharide hydrolyzing enzymes or accelerate the incorporation of soluble sugars into polysaccharides. Spd may act as a plant hormone or perhaps affect the Ca^2+^ signaling system (Alcázar et al. [2010]), participating in signal transduction under stress conditions. This effect may stimulate the synthesis and accumulation of substances that regulate osmotic adjustment in order to reduce the loss of water content of tomato seedlings and remit the damage caused by salinity-alkalinity stress.

## Conclusion

In conclusion, the present results suggested that Spd positively enhanced salinity-alkalinity tolerance in tomato. Its action was associated with nitrate metabolism, antioxidant enzymes and osmoregulation. The accumulation of compatible osmolytes may compensate for the decreased water potential during salinity-alkalinity stress. Additionally, the effect of Spd was more significantly in salt-sensitive cultivar ‘Zhongza No. 9’. Overall, exogenous Spd attenuated negative effects of saline-alkaline stress on plants which effects have varying effects on different tolerant tomato cultivars.

## Authors’ original submitted files for images

Below are the links to the authors’ original submitted files for images. Authors’ original file for figure 1 Authors’ original file for figure 2 Authors’ original file for figure 3 Authors’ original file for figure 4

## Abbreviations

CAT: Catalase
GDH: Glutamate dehydrogenase
GOGAT: Glutamine 2-oxoglutarate aminotransferase
GOT: Glutamate oxaloacetate transaminase
GPT: Glutamate pyruvate transaminase
GS: Glutamine synthetase
H_2_O_2_: Hydrogen peroxide
MDA: Malondialdehyde
NH_4_^+^: Ammonium
NH_4_^+^-N: Ammonium nitrogen
NR: Nitrate Reductase
NiR: Nitrite Reductase
NO_3_^−^: Nitrate
O_2_^.−^: Superoxide anion
POD: Peroxidase
ROS: Reactive oxygen species
SOD: Superoxide dismutase
Spd: Spermidine
