# Supplementary figures and images for: Exogenous spermidine-induced changes at physiological and biochemical parameters levels in tomato seedling grown in saline-alkaline condition
Source: Bot Stud. 2014 Aug 1;55:58. doi: 10.1186/s40529-014-0058-2 (PMC5430331; doi:10.1186/s40529-014-0058-2)

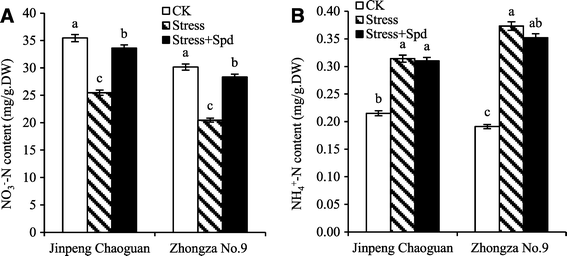

Supplement: Supplementary file 1 — Authors’ original file for figure 1 [file 40529_2014_9058_MOESM1_ESM.gif]

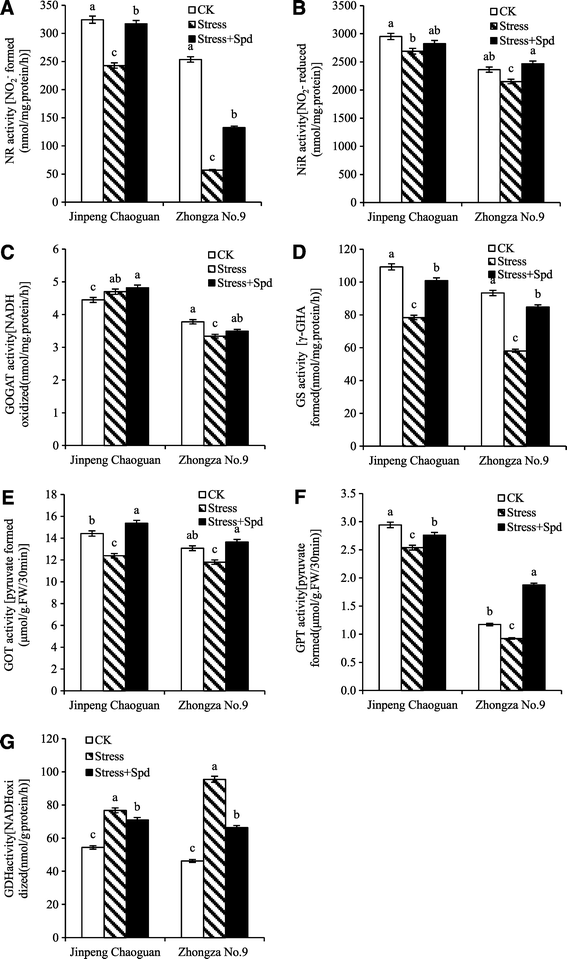

Supplement: Supplementary file 2 — Authors’ original file for figure 2 [file 40529_2014_9058_MOESM2_ESM.gif]

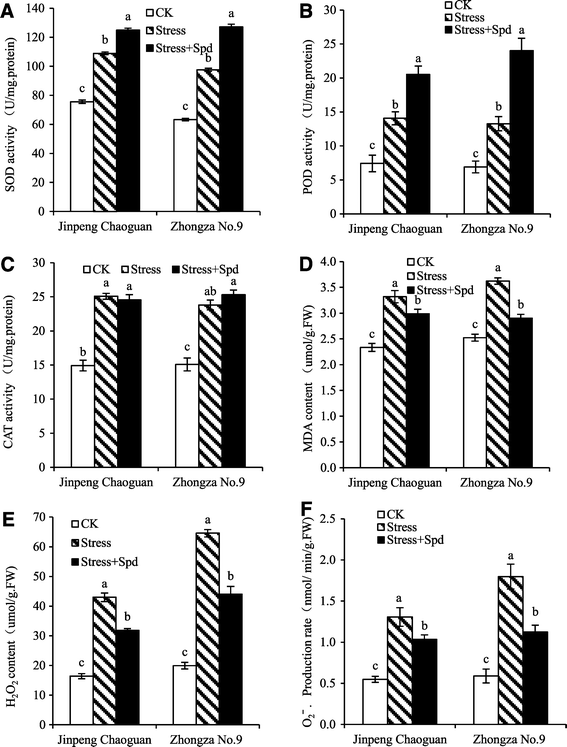

Supplement: Supplementary file 3 — Authors’ original file for figure 3 [file 40529_2014_9058_MOESM3_ESM.gif]

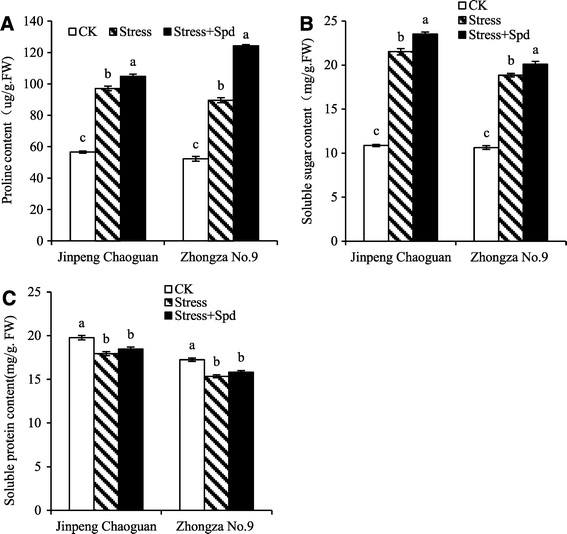

Supplement: Supplementary file 4 — Authors’ original file for figure 4 [file 40529_2014_9058_MOESM4_ESM.gif]
